# Age-related volumetric differences of hippocampal subfields in the developing brain: a retrospective MRI study

**DOI:** 10.1007/s00429-026-03074-z

**Published:** 2026-01-28

**Authors:** Sefa Işıklar, Dilek Sağlam

**Affiliations:** 1 Medical Imaging Techniques Program, Vocational School of Health Services, Bursa Uludag University, Bursa, 16240 Turkey; 2 Department of Radiology, Faculty of Medicine, Bursa Uludag University, Bursa, Turkey

**Keywords:** Hippocampal subfields, Age-related differences, Pediatrics, Volume, Asymmetry, MRI

## Abstract

Results of studies examining hippocampal subfield development and age-related differences are conflicting. This study aimed to investigate age- and sex-related volumetric differences and asymmetry in hippocampal subfields in childhood and adolescence. In this retrospective study, we included 443 individuals (200 females) with normal radiological anatomy between the ages of 2 and 18 years who had a brain MRI between 2012 and 2023. We obtained absolute and relative volumes of CA1-3, CA4-DG, and subiculum with Kulaga-Yoskovitz segmentation of volBrain HIPS in 3D-T1-weighted MRIs. We compared the volumetric data of developmental periods with SPSS (ver.28). Total hippocampal volume was consistent with quadratic models in females and sigmoid (absolute) and inverse (relative) models in males. CA1-3 showed age-related differences best characterized by sigmoid, CA4-DG by cubic and quadratic, and subiculum by cubic and power models. Total hippocampus and CA1-3 volumes were significantly smaller in the toddler period than in other periods (*p* < 0.05). While the absolute volume of the Subiculum increased significantly in all developmental periods, its relative volume increased significantly only in adolescence. CA4-DG relative volume did not differ between developmental periods (*p* > 0.05). In absolute volume, hippocampal structures differed between the sexes, but only CA4-DG had sexual dimorphism in relative volume (*p* < 0.05). In the 2–18 age group, the subiculum had left > right asymmetry, while other hippocampal subfields were lateralized to the right. This study demonstrated that hippocampal subfields exhibited heterogeneity in terms of age-related differences, with the subiculum being more sensitive to age-related factors, and CA4-DG showed a proportional increase with brain development.

**Authors**.

## Introduction

The hippocampus is a complex structure with a distinctive C-shaped appearance located in the brain’s medial temporal lobe, projecting from the floor of the temporal horn of the lateral ventricle (Insausti and Amaral 2003; Schultz and Engelhardt 2014). The hippocampus contains anatomically and functionally distinct subfields (Andersen et al. 2009). Cornu ammonis areas (CA1, CA2, CA3, and CA4), dentate gyrus (DG), and subiculum are defined as histologically distinct hippocampal subfields (Insausti and Amaral 2003; Duvernoy 2005). The hippocampus supports several important cognitive functions known to undergo significant development during childhood and adolescence (Krogsrud et al. 2014). It has been stated that the hippocampus is responsible for autobiographical memory, mental time travel (Bartsch et al. 2011), social memory (Hitti and Siegelbaum 2014), object-place learning and recall (Rolls 2013), and the encoding, storage, and retrieval of memory (Mueller et al. 2011; Senzai 2019).

The developmental trajectory and age-related differences in the hippocampus differ from those observed in other brain regions. Christova and Georgopoulos reported that (aged 6–21 years) overall cortical volume and the volumes of cortical area 30/35 decreased significantly with age, whereas hippocampal cortex volumes did not show significant age-related differences (Christova and Georgopoulos 2023). Sussman et al. reported that hippocampal relative volume increased linearly between ages 4 and 18, while subcortical gray matter regions such as the thalamus, caudate, and putamen decreased linearly (Sussman et al. 2016). Previous studies have shown that total hippocampal volume exhibits rapid growth during infancy and early childhood (Utsunomiya et al. 1999; Uematsu et al. 2012; Gilmore et al. 2012; Lee et al. 2015), followed by more modest increases in late childhood and adolescence (Uematsu et al. 2012; Hu et al. 2013; Tamnes et al. 2018). In contrast, some studies have reported that age has little or no effect on hippocampal development during adolescence (Østby et al. 2009; Muftuler et al. 2011; Koolschijn and Crone 2013).

Most studies examining the development and age-related differences of the hippocampus from childhood to adulthood have also investigated the relationship between the development of cognitive functions and the changes in hippocampal subfields (Lee et al. 2014; Keresztes et al. 2017; Riggins et al. 2018; Canada et al. 2019, 2020; Bouyeure et al. 2021). Results regarding hippocampal subfield development and age-related differences in these studies were inconsistent. Additionally, these studies do not report age-related differences in hippocampal subfields in children under the age of four. Understanding the age-related differences in hippocampal subfields, particularly during infancy, childhood, and adolescence, may facilitate anatomical-pathological distinctions.

Manual segmentation is the gold standard for assessing hippocampal subfield volumes, but it is laborious for studies with large participation (Hashempour et al. 2019). Several authors have reported that manual segmentation of hippocampal subfields required up to 20 h per subject. They also mentioned that the segmentations made require some subjectivity because they depend on the experience of the expert evaluator and are therefore open to bias (Iglesias et al. 2015; Kulaga-Yoskovitz et al. 2015). Næss-Schmidt et al. compared various hippocampal segmentation software, such as volBrain, FSL, and FreeSurfer, and reported that volBrain was superior to the other software (Næss-Schmidt et al. 2016). We therefore used volBrain, a publicly available online system for hippocampal volumetric results (Manjón and Coupé 2016; Romero et al. 2017).

A limited number of imaging studies have examined the volumetric development and age-related differences in the hippocampal formation under the age of 4 years (Utsunomiya et al. 1999; Uematsu et al. 2012; Gilmore et al. 2012; Lee et al. 2015). However, volumetric age-related differences of hippocampal subfields were not reported in these studies. Additionally, studies that included volumetric development and age-related differences in hippocampal subfields during the 2–18 age range were rare (Tamnes et al. 2014, 2018; Krogsrud et al. 2014). This study aimed to investigate whether there are age-related differences in the volume and asymmetry of hippocampal subfields using volBrain HIPS. We also identified developmental periods between the ages of 2 and 18 and compared them with other studies in similar age groups to investigate whether any differences existed. There were also conflicting reports of sex-specific differences in total hippocampal development (Suzuki et al. 2005; Uematsu et al. 2012; Hu et al. 2013; Satterthwaite et al. 2014; Wierenga et al. 2014; Krogsrud et al. 2014; Tamnes et al. 2018; Herting et al. 2018). Therefore, we also investigated the effect of sexual dimorphism on age-related differences in hippocampal subfields.

## Materials and methods

### Selection of study population

Bursa Uludağ University Faculty of Medicine Health Research Ethics Committee approved this study with the decision number 2024-11/14 dated 10.07.2024. We used some of the pediatric individuals we examined in our previous research in this study (Isıklar et al. 2022). Our study population consisted of pediatric individuals with headache, vertigo, suspected seizure-like activity, and an MRI to exclude epileptogenic pathology. We detailed the inclusion and exclusion criteria for this retrospective study in our previous study of 700 pediatric individuals (Isıklar et al. 2022). The first criterion for inclusion in the study was that individuals had a 3D-T1 sequence in their MRI protocol for the volBrain HIPS automated MRI brain volumetry system. The second criterion was that the participants’ radiological anatomy was within normal limits. The third criterion was that pediatric individuals were not followed up with any diagnosis of neurological or psychiatric disease in the radiology information system records.

Pediatric individuals between 0 and 24 months of age were not included in this study due to the low contrast of small anatomical regions such as the hippocampus in infant brain MRI and the possibility of incorrect segmentation (Makropoulos et al. 2018; Li et al. 2023). For this reason, we did not include the 140 pediatric individuals between 0 and 23.99 months that we used in our previous study in the current study (Isıklar et al. 2022). Pediatric individuals with any cerebral or cerebellar pathology other than suspected seizure-like activity and headache were excluded from the study. We excluded pediatric individuals with brain tumor, intracerebral or extracerebral hemorrhage, encephalopathy, encephalomalacia, hypomyelination, atrophy of the cerebrum and cerebellum, tuberous sclerosis, multiple sclerosis, refractory epilepsy, medial temporal sclerosis, prematurity, and those undergoing brain surgery. Kinney et al. reported that there was a risk of the hippocampal region being affected in those with a history of febrile seizures (Kinney et al. 2009). Therefore, we excluded 28 pediatric individuals with a history of febrile seizures from the study. We excluded from the study those with hippocampus images that did not fit the specified boundaries labeled by the Kulaga-Yoskovitz segmentation atlas used by volBrain HIPS1.0, without manual correction to keep the results unbiased (Kulaga-Yoskovitz et al. 2015). Therefore, we did not include 89 pediatric individuals in the study. The final sample included 443 participants (200 females and 243 males) aged 2–18 years.

### MRI scanning and segmentation of the hippocampal subfields

3D T1-weighted MR images were acquired using a Philips Achieva TX 3.0 Tesla MRI scanner (Philips Healthcare, Best, Netherlands). The parameters of the 3D-T1 weighted FFE MRI sequence of Philips 3T were as follows: The voxel size: 1 × 1 × 1 mm^3^ (isotropic), the slice thickness: 1 mm, FOV: 250 mm, TR: 8.2 ms, TE: 3.8 ms, FA: 8^0^, image matrix: 240 × 240.

**Fig. 1:**
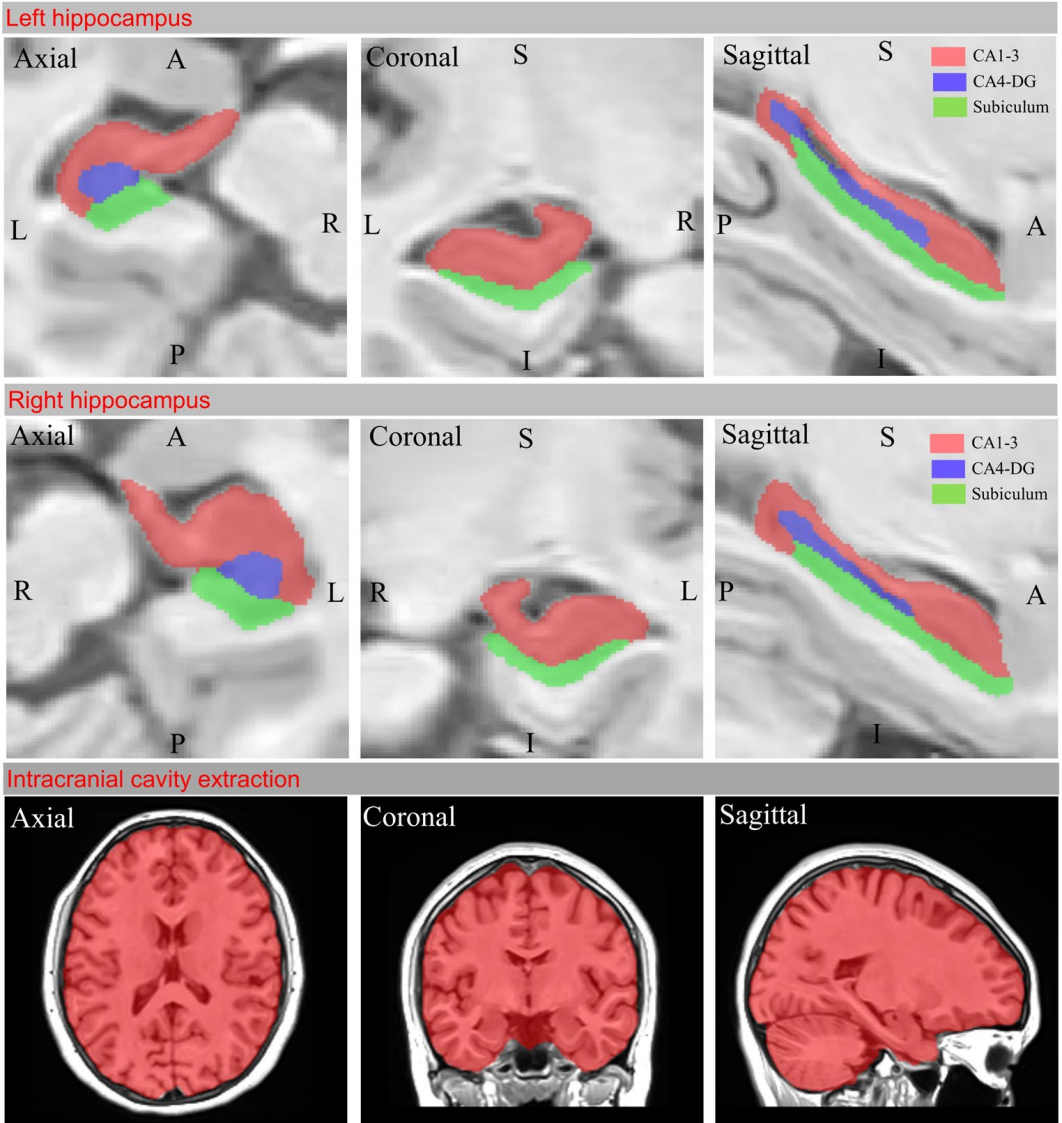
Segmentation results of cross-sectional images of hippocampal subfields and intracranial cavity extraction in MNI space (neurological orientation). Segmentation of hippocampal subfields was performed automatically by volBrain HIPS according to the protocol of Kulaga-Yoskovitz et al. (Kulaga-Yoskovitz et al. 2015). Hippocampal subfields CA (cornu ammonis)1–3 are labeled in red, CA4-DG (dentate gyrus) in blue, and the subiculum in green. The cross-sectional plane of each image is indicated in the upper-left part of the image. *R: Right*,* L: Left*,* P: Posterior*,* A: Anterior*,* S: Superior*,* I: Inferior*

**Fig. 2:**
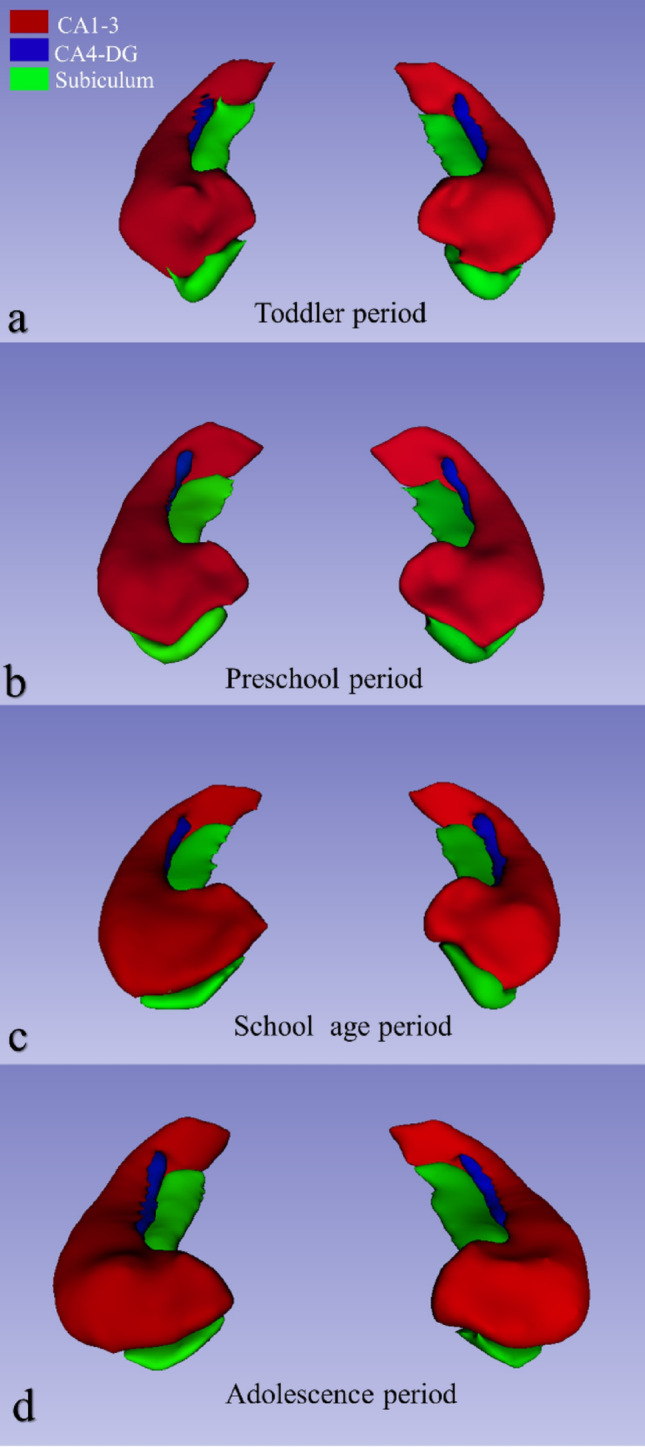
Three-dimensional (3D) view of hippocampal subfields at four different developmental periods from 2 to 18 years of age. The pediatric individuals for whom we presented 3D hippocampal subfield images were closest to the mean absolute volume of the total hippocampus during developmental stages: 30-month-old female (**a**) for the toddler period, 62-month-old female (5 years) for the preschool period (**b**), 99-month-old female (8 years) for the school age period (**c**), and 171-month-old female (14 years) for the adolescence period (**d**). Hippocampal subfields CA (cornu ammonis)1–3 are labeled in red, CA4-DG (dentate gyrus) in blue, and the subiculum in green

We used volBrain HIPS (HIPpocampus subfield Segmentation), which uses the multi-atlas-based image segmentation method, for volume measurement of the total hippocampus and hippocampal subfields (Manjón and Coupé 2016; Romero et al. 2017) (https://volbrain.net/services/HIPS). Næss-Schmidt et al. reported that volBrain had a higher dice similarity coefficient in segmenting the hippocampus than manual segmentation (Næss-Schmidt et al. 2016). For hippocampal subfield segmentation, we used the pipeline of the Kulaga-Yoskovitz in vivo high-resolution atlas of HIPS (Kulaga-Yoskovitz et al. 2015) The Kulaga-Yoskovitz atlas segments the hippocampus into CA1-3, CA4/DG, and Subiculum subfields (Fig. 1). By performing intracranial cavity extraction, volBrain HIPS calculates total intracranial volume (TIV) for relative hippocampal subfield volumes (Fig. 1). To use this pipeline, we converted the MRIs from DICOM (Digital Imaging and Communications in Medicine [.dcm]) format to NIFTI (Neuroimaging Informatics Technology Initiative [.nii]) format. We selected the HIPS1.0 pipeline from the volBrain user area. Then we selected the Kulaga-Yoskovitz atlas (3 labels) in monospectral (T1) modality. We submitted the files in NIFTI format to the software by adding sex and age information. The volBrain HIPS system automatically provides both absolute volumetric values (measured in cm³) and relative values normalized to the TIV. As stated in the volBrain HIPS report, “All the volumes are presented in absolute value (measured in cm³) and in relative value (measured in relation to the TIV)”. The normalized (relative) volumes were calculated as a percentage of each subject’s TIV according to the following formula: Normalized Volume (%TIV) = (Absolute Volume [cm³] × 100) / TIV [cm³]. These normalized (%TIV) values were used in all subsequent statistical analyses to minimize the influence of interindividual variability in head size. In hippocampal asymmetry, negative (-) asymmetric data indicate left lateralization. For quality control of automatic segmentation, we first examined the segmented labels from snapshots in the results report to evaluate deviations from the Kulaga-Yoskovitz atlas. We then examined the labeling in the axial, coronal, and sagittal planes in volBrain Preview to verify the segmentations on all cross-sectional images. We validated the accuracy of three-dimensional segmentations of hippocampal subfields using 3D Slicer (v.5.6.2) software (Fig. 2). We implemented a replica of the quality control procedure for automatic segmentation of hippocampal subfields outlined by Canada et al. (Canada et al. 2023). We excluded segmentations with missing labels, underestimated boundaries, and overestimated labels from the study without manual correction. We only manually completed some unlabeled pixels (drop-pixel errors) within the boundaries of a labeled subfield.

### Statistical analysis

We used IBM SPSS ver.28.0 software for statistical analysis (IBM Corp. Released 2021. IBM SPSS Statistics for Windows, Version 28.0. Armonk, NY: IBM Corp.). The Shapiro–Wilk test was applied to assess data normality and determine whether parametric or nonparametric tests should be used. Differences between sexes were analyzed using the Student’s t-test or the Mann–Whitney U test, as appropriate. Pearson’s or Spearman’s rank correlation coefficients were calculated to examine the strength and direction of associations between age and hippocampal volume measures.

In this study, we investigated age-related volumetric differences of hippocampal subfields in the pediatric age group using two methods. First, we examined the pediatric group in four developmental periods: Toddler (24 months – 36 months), Preschool (3–5 years), School age (6–12 years), and Adolescence (13–18 years). We showed the volume (absolute and relative) and asymmetry differences of all hippocampal subfields in females and males during developmental periods with Violin plots. Violin plots illustrating the results were created using RStudio (v. 2024.04.2, RStudio Team 2020) and the ggplot2 package (v3.5.1). In these graphs, the curved edges depict the volume distribution in the sample, and the box-shaped region in the middle of the curved edges shows the mean and quartile ranges. We also used One-Way Analysis of Variance (ANOVA) and Post Hoc Tukey HSD analysis to compare developmental periods. A p-value < 0.05 was considered statistically significant for analyses. We used the significance value (p) we created using the Bonferroni correction as the threshold value for multiple comparisons between the developmental periods of hippocampal subfields.

Second, using Python, we created volumetric distribution plots of hippocampal subfields between ages 2 and 18. Graphs were performed using Python (version 3.12) and the packages numpy (2.0), pandas (2.2.2), scipy (1.14.0), matplotlib (3.9.1), and Pyreadstat (1.2.7). We examined developmental models using linear and polynomial regression analyses and used the best-fit slope plot to identify age-related relationships best.

## Results

### Participant characteristics and total intracranial volume

To understand the age-related differences of the hippocampus, we first characterized our study population of 443 individuals aged 2 to 18 years (mean age: 8.21 ± 5.17 years). The cohort consisted of 200 females (45.1%) with a mean age of 8.58 ± 5.34 years and 243 males (54.9%) with a mean age of 7.90 ± 5.01 years. An initial analysis aimed to determine if age distributions differed between sexes, which could confound developmental results. We found no significant difference in age between males and females in the total population or within specific developmental periods (*p* > 0.05, Table 1).

For a more detailed analysis of age-related differences, we categorized participants into four periods: toddlers (24-35.99 months; *n* = 55, 27 female; mean age: 29.98 ± 3.55 months), preschool (36-71.99 months; *n* = 127, 53 female; mean age: 51.98 ± 10.33 months), school-age (72-155.99 months; *n* = 141, 56 female; mean age: 107.16 ± 23.28 months), and adolescence (156-227.99 months; *n* = 120, 64 female; mean age: 193.13 ± 20.66 months).

We also analyzed total intracranial volume (TIV) to determine how total brain size changes across these periods. TIV for the entire 2–18 year age range was 1319.85 ± 149.18 cm³. As expected, TIV increased significantly with age; TIV in the toddler and preschool periods was significantly smaller than in the school-age and adolescent periods (*p* < 0.0083). However, we did not detect a significant difference in TIV between the school-age and adolescent periods (*p* > 0.0083, Table 1), suggesting that much of the increase in overall brain size occurs before the school-age years.

### Age and sex-based volumetric differences in the total hippocampus

The primary question of this study was to determine whether the hippocampus exhibits age- and sex-related differences from early childhood to late adolescence. To address this, we examined both absolute and relative hippocampal volumes. Absolute volume analysis allows characterization of age-related differences in physical structure. In contrast, relative volume analysis (normalized by TIV) clarifies whether these differences are proportional to or distinct from overall brain development.

The mean absolute and relative volumes of the total hippocampus in the 2–18 age group were 6.40 ± 0.85 cm³ and 0.49 ± 0.04%, respectively, with a rightward asymmetry (right > left, 3.67 ± 4.26%). Correlation analysis indicated a moderate positive correlation between absolute hippocampal volume and age (*r* = 0.453; *p* < 0.001), but only a low positive correlation for relative volume (r: 0.212; *p* < 0.001). This indicates that while the hippocampus grows with age, much of this growth is proportional to the overall increase in brain size. Hippocampal asymmetry was not significantly correlated with age (*p* > 0.05).

Comparing across developmental periods, ANOVA revealed significant differences for all volumetric data except asymmetry (*p* < 0.05). Post-hoc Tukey HSD tests showed significant increases in absolute volume between all periods (*p* < 0.0083), except between the school-age and adolescence periods (*p* > 0.0083, Table 2). In contrast, a significant difference in relative volume was found only between the toddler and adolescent periods (*p* < 0.0083, Table 2), further indicating that hippocampal growth relative to total brain size is most pronounced early in development.

**Fig. 3:**
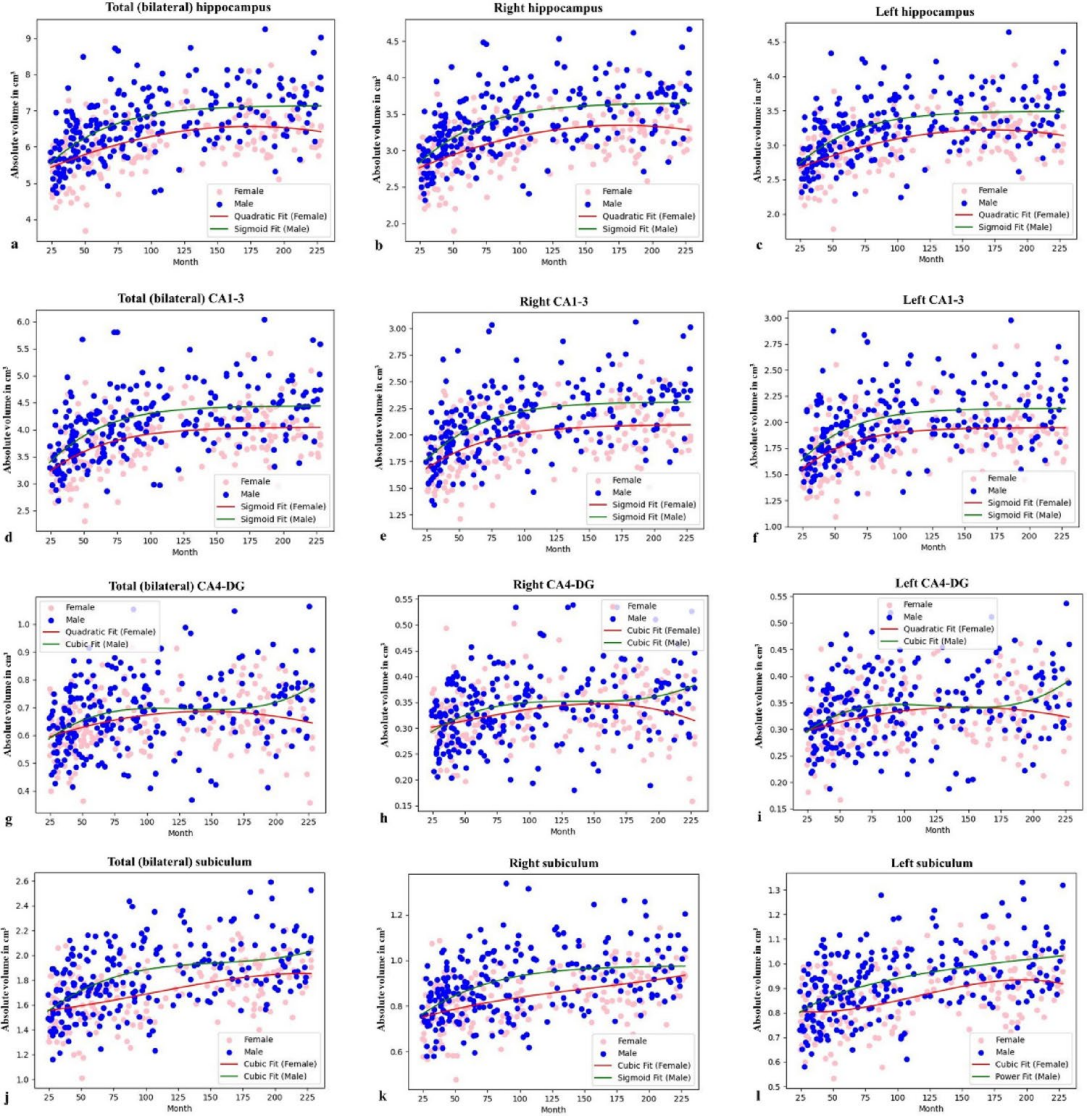
Python-generated scatter plots of absolute volumetric data of total hippocampus and hippocampal subfields in males and females. The curves of the models that best represent the age- and sex-related variation in the absolute volume of the total hippocampus (**a**: bilateral; **b**: right; **c**: left), CA1-3 (**d**: bilateral; **e**: right; **f**: left), CA4-DG (**g**: bilateral; **h**: right; **i**: left) and subiculum (**j**: bilateral; **k**: right; **l**: left) were shown by matching them with scatter plots. The R^2^, F, and p values of the age-related models in these graphs are given in Table 3. *CA: cornu ammonis*,* DG: dentat girus*

**Fig. 4:**
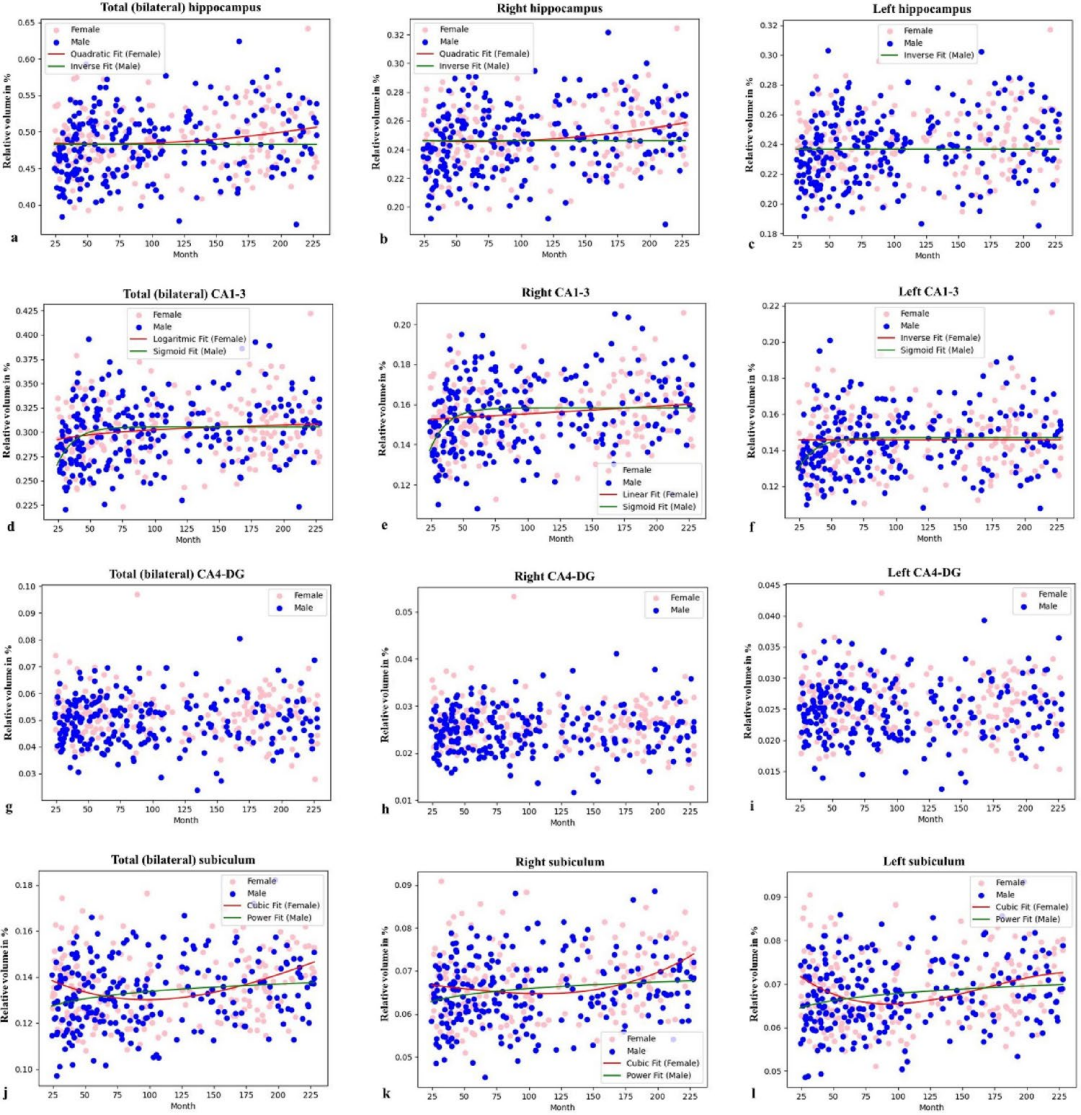
Python-generated scatter plots of relative volumetric data of total hippocampus and hippocampal subfields in males and females. The curves of the models that best show the age- and sex-related differences in the relative volume of the total hippocampus (**a**: bilateral; **b**: right; **c**: left), CA1-3 (**d**: bilateral; **e**: right; **f**: left), CA4-DG (**g**: bilateral; **h**: right; **i**: left) and subiculum (**j**: bilateral; **k**: right; **l**: left) were shown by matching them with scatter plots. The R^2^, F, and p values of the age-related models in these graphs are given in Table 3. *CA: cornu ammonis*,* DG: dentat girus*

To investigate sex-specific age-related differences, we performed a regression analysis. This revealed distinct age-related models between females and males. The absolute volume of the total hippocampus was best described by a quadratic model in females and a sigmoid model in males (Fig. 3; Table 3). The quadratic model in females suggests a nonlinear age-related difference that accelerates in childhood and decelerates in adolescence. In contrast, the sigmoid model in males indicates a more distinct age-related difference onset, followed by a rapid increase and then a plateau. For relative volume, females followed a quadratic model, while males exhibited a reverse model (Fig. 4; Table 3), the latter suggesting that the relative proportion of the hippocampus may decrease over time due to a more substantial increase in total brain volume in males.

**Fig. 5:**
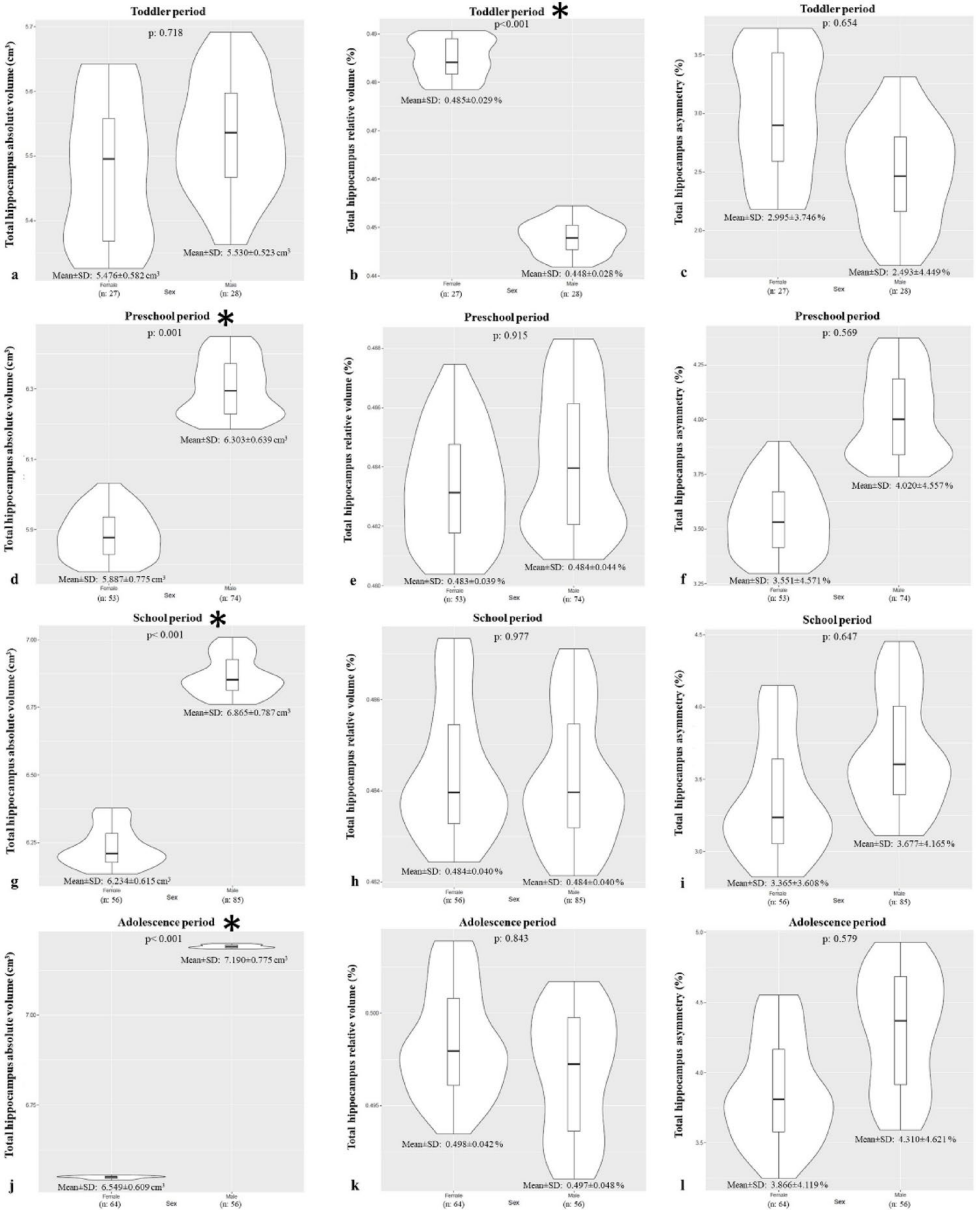
Violin plots created with RStudio showing the volume (absolute and relative) and asymmetry differences of the total hippocampus in females and males during toddler (**a**, **b**, **c**), preschool (**d**, **e**, **f**), school age (**g**, **h**, **i**) and adolescence (**j**, **k**, **l**) periods. The curved edges depict the volume distribution in the sample, and the box plots show the mean and quartile ranges. Asterisks indicate developmental periods during which the total hippocampus differed between sexes (*p* < 0.05)

Direct comparisons showed that between 2 and 18 years, the absolute volume of the total hippocampus was significantly larger in males (6.62 ± 0.88 cm³) than in females (6.14 ± 0.75 cm³) (*p* < 0.05). This difference was significant across all developmental periods except the toddler stage (*p* < 0.05, Fig. 5) However, there was no overall sexual dimorphism in relative volume (female: 0.489 ± 0.039%; male: 0.483 ± 0.044%; *p* > 0.05). A significant difference in relative volume was observed only during the toddler period, where females had a greater relative volume than males (*p* < 0.05, Fig. 5) Compared with the toddler period, the mean absolute volume of the total hippocampus increased by 11.38%, 20.20%, and 24.45% in the preschool, school-age, and adolescent periods, respectively. The increase in mean relative volume was more modest at 3.80%, 3.90%, and 6.76%, respectively.

### Age-related models of hippocampal subfields

To understand how the volumes of hippocampal subfields change with age, this study investigated their specific age-related models. For the CA1-3 subfield, absolute volume showed a moderate positive correlation with age (*r* = 0.414; *p* < 0.001), and relative volume had a weak positive correlation (*r* = 0.189; *p* < 0.001). Regression analysis revealed a sigmoid model for absolute volume in both sexes (Fig. 3; Table 3). For relative volume, females followed a logarithmic model, while males showed a sigmoid model (Fig. 4; Table 3), with higher R² values in males for both volume types, suggesting more dynamic age-related differences.

In the CA4-DG subfield, only the absolute volume showed a low positive correlation with age (*r* = 0.236; *p* < 0.001). Regression models identified a quadratic model for absolute volume in females and a cubic model in males (Fig. 3; Table 3); however, no significant model was found for relative volume in either sex (*p* > 0.05, Fig. 4; Table 3).

Lastly, the subiculum’s absolute volume demonstrated a moderate positive correlation with age (*r* = 0.430; *p* < 0.001), while its relative volume showed a low correlation (*r* = 0.181; *p* < 0.001). Regression analysis indicated a cubic model for absolute volume in both sexes (Fig. 3; Table 3). For relative volume, females followed a cubic model, and males a power one (Fig. 4; Table 3). Asymmetry showed no correlation with age for any subfield (*p* > 0.05).

### Sex-based volumetric differences in hippocampal subfields

**Fig. 6:**
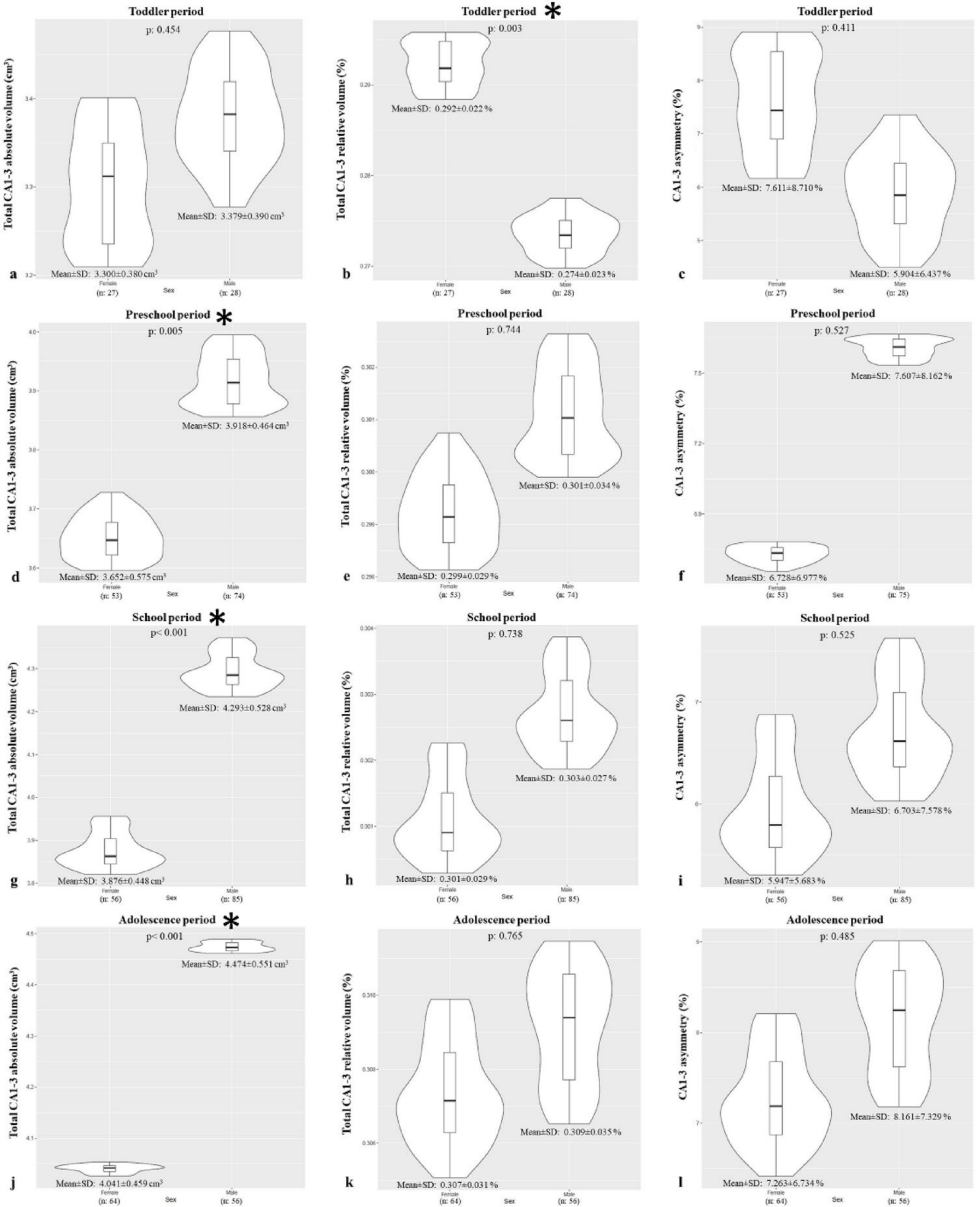
Violin plots created with RStudio showing the volume (absolute and relative) and asymmetry differences of CA (cornu ammonis)1–3 in females and males during toddler (**a**, **b**, **c**), preschool (**d**, **e**, **f**), school age (**g**, **h**, **i**) and adolescence (**j**, **k**, **l**) periods. The curved edges depict the volume distribution in the sample, and the box plots show the mean and quartile ranges. Plots with asterisks indicate developmental periods during which CA1-3 showed sexual dimorphism (*p* < 0.05)

This analysis sought to determine if volumetric differences exist between males and females across the hippocampus and its subfields. Overall, the absolute volume of the CA1-3 was significantly larger in males (4.12 ± 0.60 cm³) than in females (3.79 ± 0.54 cm³) (*p* < 0.05), a difference that was significant in all developmental periods except the toddler stage (*p* < 0.05, Fig. 6). While there was no overall sex difference in relative CA1-3 volume (*p* > 0.05), females had a significantly greater relative volume than males during the toddler period (*p* < 0.05, Fig. 6).

**Fig. 7:**
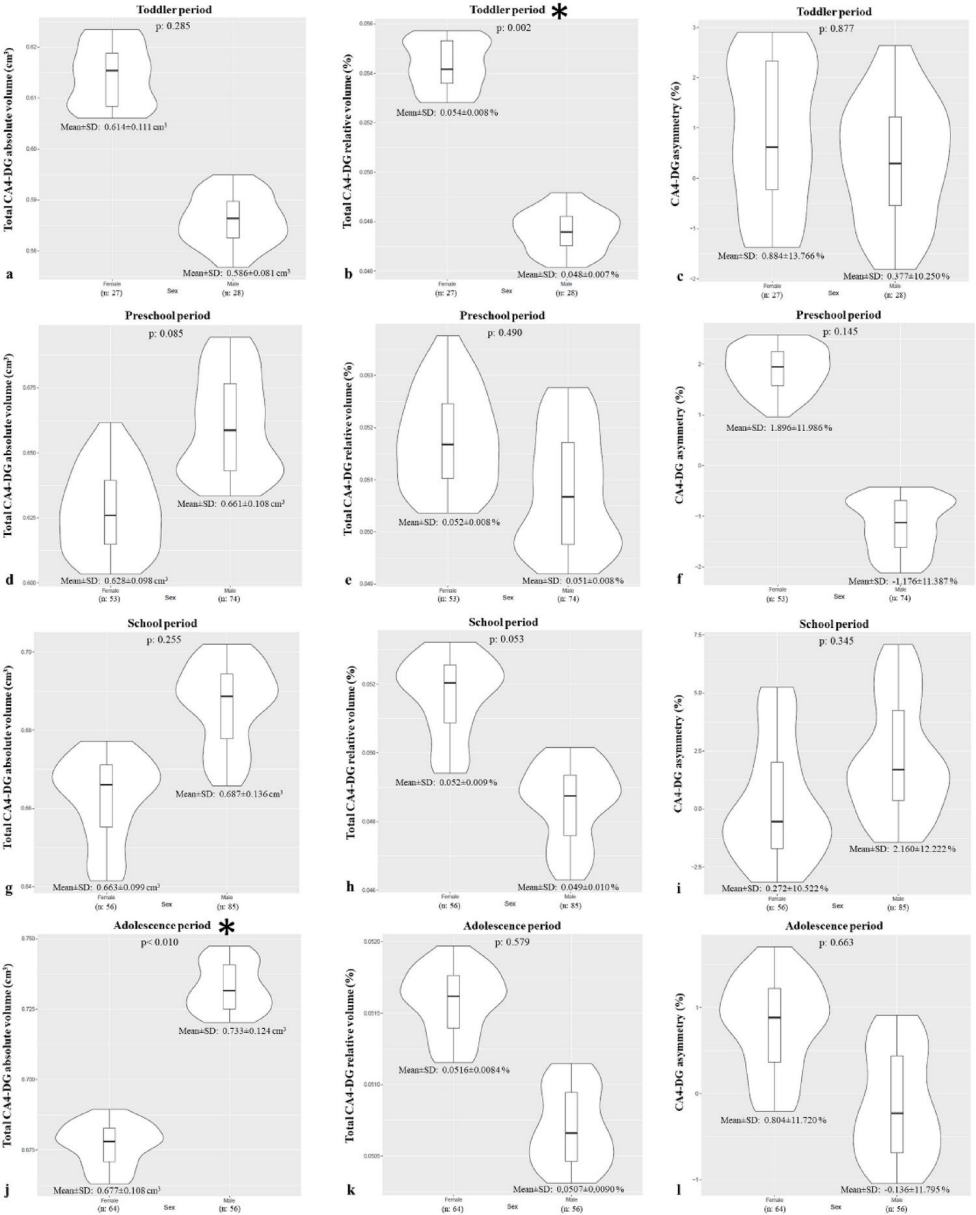
Violin plots created with RStudio showing the volume (absolute and relative) and asymmetry differences of CA4-DG in females and males during toddler (**a**, **b**, **c**), preschool (**d**, **e**, **f**), school age (**g**, **h**, **i**) and adolescence (**j**, **k**, **l**) periods. The curved edges depict the volume distribution in the sample, and the box plots show the mean and quartile ranges. Plots with asterisks indicate developmental periods during which CA4-DG showed sexual dimorphism (*p* < 0.05). *CA: cornu ammonis*,* DG: dentat girus*

For the CA4-DG, the absolute volume was larger in males (0.34 ± 0.07 cm³) than in females (0.33 ± 0.06 cm³) (*p* < 0.05), while the relative volume was significantly larger in females (0.052 ± 0.008%) than in males (0.050 ± 0.009%) (*p* < 0.05). When examined by period, the absolute volume was larger in males only during adolescence (*p* < 0.05, Fig. 7), and the relative volume was larger in females only during the toddler period (*p* < 0.05, Fig. 7).

**Fig. 8:**
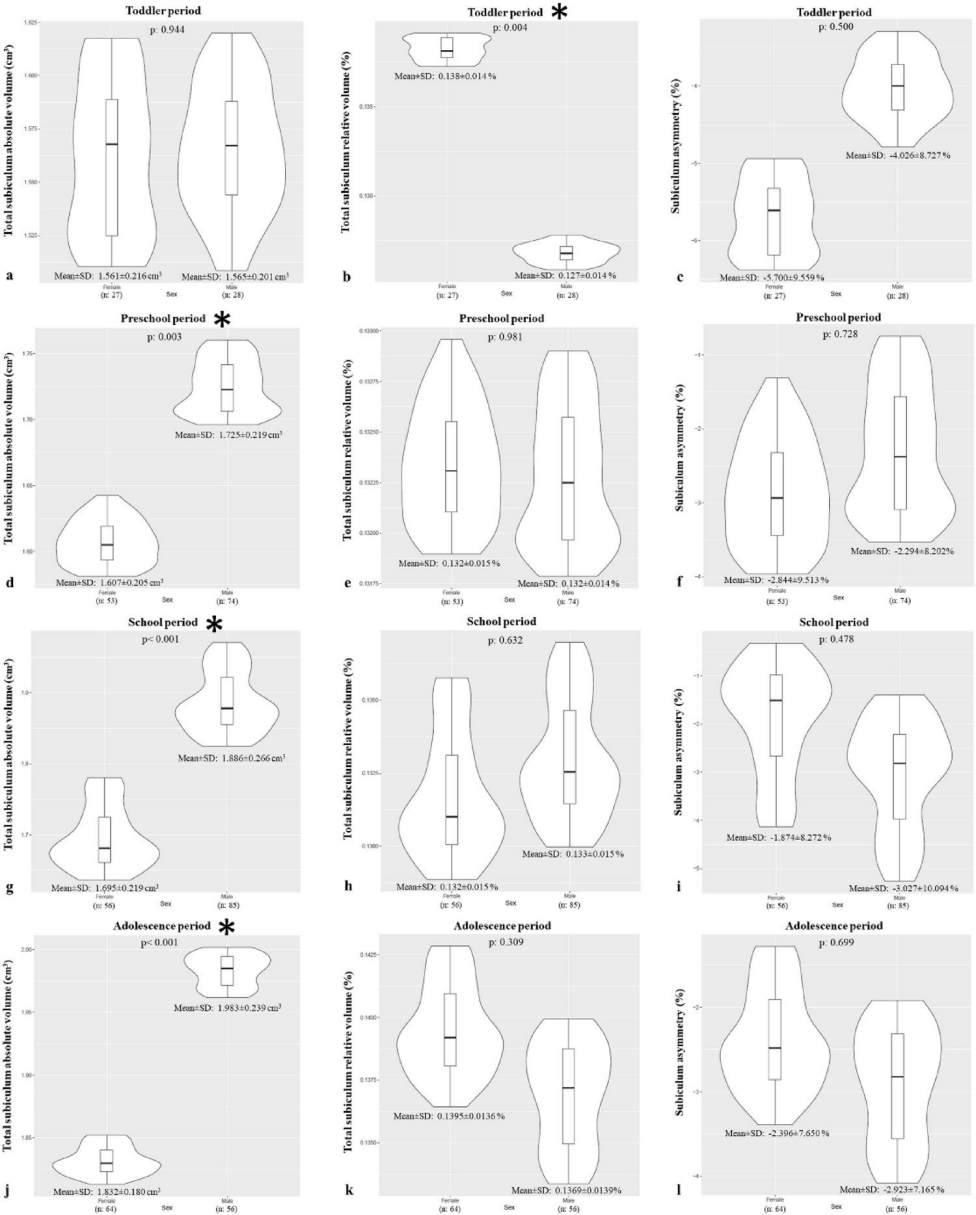
Violin plots created with RStudio showing the volume (absolute and relative) and asymmetry differences of the subiculum in females and males during the toddler (**a**, **b**, **c**), preschool (**d**, **e**, **f**), school age (**g**, **h**, **i**) and adolescence (**j**, **k**, **l**) periods. The curved edges depict the volume distribution in the sample, and the box plots show the mean and quartile ranges. Plots with asterisks indicate developmental periods during which subiculum showed sexual dimorphism (*p* < 0.05)

Similarly, the absolute volume of the subiculum was significantly larger in males (1.82 ± 0.27 cm³) than in females (1.70 ± 0.23 cm³) across the entire age range (*p* < 0.05), with this difference being significant in all periods except the toddler stage (*p* < 0.05, Fig. 8). There was no overall difference in the subiculum’s relative volume (*p* > 0.05), but mirroring the other subfields, it was significantly larger in females than males during the toddler period (*p* < 0.05, Fig. 8).

### Differences in hippocampal subfields based on developmental period

To determine if volumetric changes occur in distinct stages, this study compared hippocampal subfield volumes across four developmental periods. ANOVA confirmed significant volumetric differences for the CA1-3 subfield across periods (*p* < 0.05), where the absolute volume in the toddler and preschool periods was significantly different from the school-age and adolescence periods (*p* < 0.0083, Table 2). For relative CA1-3 volume, a significant difference was found only between the toddler period and the other periods (*p* < 0.0083, Table 2). From the toddler period, the mean absolute volume of the total CA1-3 increased by 13.98%, 23.57%, and 27.04% in the subsequent periods, with relative volume increases of 6.21%, 6.83%, and 8.99%.

For the CA4-DG, ANOVA indicated a significant difference across periods only for absolute volume (*p* < 0.05, Table 2), with post-hoc tests revealing that the adolescence period had a significantly larger absolute volume than the toddler and preschool periods (*p* < 0.0083, Table 2). Compared to the toddler period, the mean absolute volume of the CA4-DG increased by 7.84%, 12.88%, and 17.19% across the subsequent periods, while mean relative volumes showed minimal changes of 0.54%, -2.27%, and 0.47%.

In the subiculum, ANOVA revealed significant differences for absolute volume (*p* < 0.05), with post-hoc tests showing that the toddler and preschool periods were significantly different from the school-age and adolescence periods (*p* < 0.0083, Table 2) From the toddler baseline, the subiculum’s mean absolute volume increased by 7.19%, 15.80%, and 21.70% in the subsequent periods, with corresponding changes in mean relative volumes of -0.09%, 0.08%, and 4.41%. Asymmetry showed no significant differences across developmental periods for any subfield.

## Discussion

This study detailed the age-related differences in the total hippocampus and its subfields (CA1–3, CA4–DG, and subiculum) concerning absolute and relative volumes during the pediatric period. All hippocampal subfields showed a statistically significant absolute volume increase with age. The fact that the age-related models were mostly in sigmoid, cubic, or quadratic forms indicates that the increase was not linear and varied across different age ranges. Relative volume analyses revealed no significant age-related differences, particularly in the CA4-DG regions, indicating that these regions scale proportionally with overall brain size and remain relatively stable across age. On the other hand, sex-specific age-related differences were observed in the CA1–3 and subiculum subfields (e.g., cubic and quadratic models were more frequent in females, whereas sigmoid models were more common in males). This suggests that age-related differences in hippocampal subfields may be closely related to sex. CA1-3, CA4-DG had right > left asymmetry, while the subiculum had left > right lateralization. There was no sexual dimorphism in the lateralization of hippocampal subfields.

Interest in the developmental plasticity of the hippocampus has increased with software that automatically segments high-resolution MR images. However, there is still no consensus on how the hippocampus and its subfields are affected by age. Unlike other studies, we examined age-related volumetric differences in the total hippocampus and its subfields across four age ranges between 2 and 18 years. This was to detect subtle signs of development in the hippocampus and contribute to the volumetric reference data.

### Age-related differences in the total hippocampus

Lee et al. (2–4 years of age) and Uematsu et al. (first 4–5 years of life) found age-related increases in hippocampal volume, consistent with this study (Uematsu et al. 2012; Lee et al. 2015). Uematsu et al. reported that absolute volume increases in both hippocampi from infancy to preadolescence (9–11 years of age) had nonlinear age-related differences similar to our study (Uematsu et al. 2012). Suzuki et al. reported that healthy males in late adolescence had significantly larger hippocampal volumes than those in early adolescence (Suzuki et al. 2005). Recent longitudinal studies have reported that postnatal development and age-related differences in the hippocampus are characterized by a positive increase in total volume throughout childhood and adolescence (Wierenga et al. 2014; Tamnes et al. 2018; Canada et al. 2020). In this study, differences in hippocampal absolute volume were found between toddlerhood and other childhood periods (preschool and school-age periods) and adolescence. Although there was a quantitative increase between school age and adolescence periods, we did not detect any significant difference between the periods. In terms of relative volume, the toddler period and adolescence differ from other periods. However, it was surprising that there was no significant difference between the preschool and school age periods. For these reasons, this study is consistent with studies that explain the continuing volumetric increase in the hippocampus throughout childhood with a nonlinear age-related model (Uematsu et al. 2012; Krogsrud et al. 2014).

### Methodological challenges in comparing studies of developmental and age-related differences in hippocampal subfields

Until recently, the hippocampus was considered as a whole in neuroimaging studies. Assessing hippocampal volume as a whole may not accurately reflect developmental and age-related differences because it overlooks the heterogeneous infrastructure of the hippocampus. Different structural areas of the hippocampus based on its cytoarchitecture have recently become the focus of attention. Studies on age-related change in the hippocampus during childhood and adolescence have focused on the relationships between different hippocampal subregions and various cognitive functions (Lee et al. 2014; Riggins et al. 2018; Canada et al. 2019; Bouyeure et al. 2021). However, these studies were not conducted on all hippocampal subfields, but on subfields in the head and body regions of the hippocampus. We believe that body region-specific studies have increased due to studies showing that the volume of the hippocampal head decreases and the hippocampal body increases with age (Gogtay et al. 2006; Master et al. 2014). Additionally, identifying hippocampal subfields is difficult in the head and tail compared to the body. Examining a specific part of the anteroposterior axis (hippocampal subregions) rather than the entire hippocampal subfields may affect developmental outcomes (Master et al. 2014). Additionally, it was difficult to directly compare the evidence from these reports because of the differences in age range across these studies and the different segmentation methods they used. The two studies closest to the age group of this study were the cross-sectional study by Krogsrud et al. (4–22 years) and the longitudinal studies by Tamnes et al. (8–19 years and 8–26 years) (Tamnes et al. 2014, 2018; Krogsrud et al. 2014). Both authors used FreeSurfer version 5.1 and version 6.0 for hippocampal subfield segmentation. Schoene-Bake et al. reported that FreeSurfer version 5.1 systematically misestimated certain hippocampal subregion volumes (Schoene-Bake et al. 2014). Recently, Özen et al. examined changes in hippocampal subfields in 138 healthy individuals aged 4 to 88 using volBrain HIPS (Özen et al. 2025). Unlike the study by Özen et al., we focused on the pediatric period (2–18 years) in this study. Therefore, this study was the first to examine the age-related fine differences of hippocampal subfields in the developing brain with volBrain HIPS.

Our study used a cross-sectional design, which allows us to examine age-related differences in hippocampal subfield volumes across participants at a single time point. However, longitudinal evidence indicates that cross-sectional findings may not directly reflect within-person developmental trajectories. Keresztes et al. showed that cross-sectional age associations in hippocampal subfields do not necessarily align with longitudinal developmental changes, highlighting that inferences about neural development from cross-sectional data should be made cautiously (Keresztes et al. 2022).

Keresztes et al. reported that cross-sectional age-associations at baseline (Wave 1) and follow-up (Wave 2) suggested increases in CA1-2 and DG-CA3 volumes, but their longitudinal latent change score models showed no significant mean increase in these subfields (Keresztes et al. 2022). Instead, they observed significant increases only in subiculum and entorhinal cortex volumes. The subiculum consistently emerged as a key region in both studies; our study demonstrated a strong relationship with age, while their longitudinal study provided concrete evidence for both volumetric increase and its link to spatial memory development.

Due to difficulties in image acquisition and segmentation processes in early childhood, our knowledge about the development of hippocampal subfields remained limited. Studies on hippocampal subfields under the age of four were related to the effects of cognitive activities and various diseases on the hippocampus (Li et al. 2019; Ellis et al. 2021; Mason and Spencer 2022). However, to our knowledge, no volumetric investigation of age-related differences in hippocampal subfields spanning the toddler period has been conducted. We believe this study will increase our understanding of age-related differences in hippocampal subfields between 24 and 36 months.

### Age-related differences in the CA1-3 subfields

Consistent with the findings of Krogsrud et al., this study observed a positive correlation between age and the volume of the CA1-3 subfields of the entire hippocampus (Krogsrud et al. 2014). We identified a volumetric increase in this region up to age 18, with the largest absolute and relative volumes present during adolescence. This aligns with a longitudinal study by Tamnes et al., which reported a significant increase in CA1 volume from age 8 to approximately age 20, followed by a slight decrease (Tamnes et al. 2018). However, not all studies report positive growth. Some have documented a negative association with age and volumetric decrease in childhood and adolescence (Tamnes et al. 2014; Schlichting et al. 2017). These discrepancies may be attributable to differences in the specific CA subfields analyzed (e.g., CA1 vs. CA2-3), the portion of the hippocampus examined (e.g., head, body, or tail), and the age range of the participants. For example, Bouyeure et al. found no significant relationship between age and hippocampal growth in CA2-3 in the hippocampal body during childhood (Bouyeure et al. 2021). Additionally, Schlichting et al. found no significant relationship between age and hippocampal growth in CA2-3 in the hippocampal head and body between 6 and 30 years of age (Schlichting et al. 2017). Taken together, the evidence suggests a complex, nonlinear age-related model for the CA1-3 subfields, with our findings supporting a prolonged period of volumetric increase that peaks in adolescence.

The observed sigmoid model of absolute volume in the CA1-3 subfield for both sexes aligns with previous work suggesting a period of growth followed by stabilization during adolescence (Krogsrud et al. 2014; Dick et al. 2022). For instance, Krogsrud and colleagues reported rapid age-related increases in most hippocampal subfields until approximately 13 to 15 years of age (Krogsrud et al. 2014). The sex-specific models for relative CA1-3 volume (logarithmic for females and sigmoid for males) suggest a more prolonged and gradual maturational process in females than a more dynamic, growth-spurt-like pattern in males. This aligns with the broader trajectory of sex differences in brain development, where males sometimes exhibit more pronounced and later-peaking changes (Fish et al. 2020; Mu et al. 2020).

The analysis by discrete developmental periods provides a granular view that complements the continuousage-related models. The finding that absolute volumes in the school-age and adolescent periods were significantly larger than in earlier periods confirms that the transition out of early childhood marks a major phase of structural growth. The magnitude of this change is substantial, with a mean increase of over 27% in absolute volume from the toddler years to adolescence. This period of intense growth coincides with a critical window for the development of complex memory abilities, which are heavily dependent on the CA subfields (Keresztes et al. 2017). Interestingly, for relative volume, the only significant difference was observed between the toddler period and all subsequent periods. This suggests that after an initial phase of disproportionate growth in early life, the maturation of the CA1-3 subfield proceeds largely in proportion to overall brain growth.

### Age-related differences in the CA4–DG subfields

A consensus on the developmental course and age-related differences of the CA4-DG has been elusive in the literature. Our findings are in general agreement with cross-sectional studies that report increases in dentate gyrus (DG) volume from age 4 through childhood and adolescence (Krogsrud et al. 2014). The growth trend observed in the work by Krogsrud and colleagues aligns with the ascending portion of the quadratic trajectory we observed in females and the overall positive trend of the cubic model in males (Krogsrud et al. 2014). However, these findings are in stark contrast to influential longitudinal studies by Tamnes et al., which consistently report a linear decrease in CA4-DG volume from age eight throughout adolescence (Tamnes et al. 2014, 2018). The volumetric decline observed in these longitudinal studies has often been attributed to maturational processes such as synaptic pruning and neural circuit reorganization (Tamnes et al. 2018). The variability in findings becomes even more apparent in studies examining specific developmental segments; for instance, Bouyeure et al. found no significant relationship between age and CA4-DG volume in the hippocampal body during childhood (Bouyeure et al. 2021). The complex, non-linear models identified in our study suggest that the age-related differences of the CA4-DG may encompass periods of both volumetric increase and stabilization or decline. This could explain why studies using different age ranges or methodologies (cross-sectional vs. longitudinal) might capture different segments of the trajectory, thereby reporting conflicting results.

One of the most notable findings of our study is that despite the significant and complex models observed for absolute volume, no significant model was detected for relative volume in either sex (Fig. 4). This suggests that the volumetric differences of the CA4-DG proceed largely in proportion to overall brain growth. In other words, rather than exhibiting disproportionate growth or atrophy independent of other brain structures, the CA4-DG appears to develop in concert with global brain maturation. This may somewhat challenge the hypothesis that significant synaptic pruning leads to a volumetric decline, as such a process might be expected to also cause a decrease in relative volume (Tamnes et al. 2014).

### Age-related differences in the subiculum

Our primary finding of a moderate positive correlation between absolute subiculum volume and age is broadly consistent with a substantial portion of the cross-sectional literature, which has repeatedly documented volumetric increases throughout childhood and adolescence (Krogsrud et al. 2014; Canada et al. 2019, 2021; Bouyeure et al. 2021). Schlichting et al.‘s finding that subiculum volume was negatively correlated with age contradicts these reports’ finding of an increase (Schlichting et al. 2017). However, the identification of a cubic age-related model for absolute volume in both sexes suggests a more complex relationship of age-related difference than a simple linear increase. This cubic model, which included multiple phases of acceleration and deceleration in growth, contradicted previous reports that subiculum volume was invariant with age (Lee et al. 2014; Daugherty et al. 2017; Riggins et al. 2018). The cubic model observed in our data may reflect an initial rapid growth spurt in early childhood, followed by a period of sustained growth through the school-age years, and finally a slowing or plateauing as adolescence progresses. This model helps reconcile some of the most striking discrepancies in the literature, particularly those related to longitudinal findings. For instance, while one longitudinal study reported a surprising linear decrease in subiculum volume (Tamnes et al. 2014), a subsequent, larger study by the same group found a non-linear pattern of increase until mid-adolescence, followed by a subtle decline (Tamnes et al. 2018). Our cross-sectional cubic model supports this latter view of a protracted and dynamic growth period rather than a simple decrease.

The analysis by developmental periods further illuminates this complex trajectory. The significant increase in absolute subiculum volume from the toddler and preschool periods to the school-age and adolescent periods highlights a major maturational leap occurring after early childhood. The substantial increase of 21.70% in mean absolute volume from the toddler period to adolescence quantifies the extensive growth this structure undergoes. Even more telling is the analysis of relative volume. While the correlation with age was low, the presence of significant, non-linear models (cubic for females, power for males) indicates that the subiculum’s growth is not merely proportional to overall brain maturation. The notable 4.41% increase in mean relative volume specifically during adolescence suggests that this period is a time of disproportionate growth and specialization for the subiculum. This late-stage maturation likely reflects ongoing microstructural changes, such as protracted myelination and synaptic reorganization, which are critical for refining the hippocampal-cortical circuits that support higher-order cognitive functions (Keresztes et al. 2017).

### Sexual dimorphism

This study was consistent with studies reporting that males have larger volumes than females in hippocampal development (Giedd et al. 2012; Uematsu et al. 2012; Hu et al. 2013; Krogsrud et al. 2014; Tamnes et al. 2018). However, we found no significant difference between sexes in the total hippocampus to TIV ratio, except in the toddler period (females had larger relative hippocampal volume than males). Herting et al. reported that females have larger hippocampi than males between the ages of 8 and 10 (Herting et al. 2018). Satterthwaite et al. found no sexual dimorphism in prepubertal hippocampal volumes but significantly larger bilateral hippocampal volumes in postpubertal females (Satterthwaite et al. 2014). Unlike Satterthwaite et al., this study found greater hippocampal volume in males during adolescence.

Wierenga et al. found greater total hippocampal volume in males between 7 and 23 years of age but no sexual dimorphism in hippocampal developmental trajectory (Wierenga et al. 2014). Unlike Wierenga et al., this study found sex differences in total hippocampal age-related models in males (absolute: sigmoid; relative: inverse) and females (absolute: quadratic; relative: quadratic).

Consistent with previous literature, males exhibited significantly larger absolute volumes across the CA1-3, CA4-DG, and subiculum subfields from the preschool period onward (Tamnes et al. 2014; Krogsrud et al. 2014). However, our analysis of relative volumes and models of age-related differences revealed a more nuanced picture that diverges from prior reports. Specifically, a novel finding emerged during early development: females displayed significantly greater relative volumes in the CA1-3, CA4-DG, and subiculum subfields during the toddler period, suggesting a potential for earlier structural maturation or a different scaling relationship with total intracranial volume in young females. Furthermore, we identified fundamentally different age-related dynamics between sexes, a finding that contrasts with earlier studies suggesting no sexual dimorphism in age-related models (Tamnes et al. 2018). For instance, the relative volume of CA1-3 was best described by a logarithmic model in females and a sigmoid model in males, while the subiculum followed cubic and power model in females and males, respectively. This divergence in maturational timing and dynamics may represent a neural substrate for sex-specific differences in the development of learning and memory strategies.

### Asymmetry

Pfluger et al. found that the right hippocampus was on average 0.10 cc larger in normally developing children in a similar age range to our study (1 month to 15 years) (Pfluger et al. 1999). This study was consistent with studies that found the total hippocampus right-lateralized without sex differences (Uematsu et al. 2012; Tamnes et al. 2014; Krogsrud et al. 2014). Thompson et al. stated that the reason they found the hippocampus to be right lateralized without any sex difference could be that babies need visual-spatial abilities (right hippocampus function) more than their linguistic abilities (left hippocampus function) (Thompson et al. 2009). Krogsrud et al. found that the volumes of the right CA1, CA2/3, and CA4/DG and the left subiculum were larger than those of the similar hippocampal subfield in the contralateral hemisphere, and this result was consistent with our study (Krogsrud et al. 2014). Özen et al. examined the change in asymmetry in hippocampal subfields between the ages of 4 and 88 with volBrain HIPS (Özen et al. 2025). They reported that, similar to our study, the number of individuals with right > left asymmetry in CA1-3 and those with left > right asymmetry in the subiculum were significantly higher. Moghaddam et al. found similar hippocampal subfield lateralization as in this study in CA2-3 (15.16%) and subiculum (-7.99%) with volBrain HIPS in healthy mature hippocampus (Moghaddam et al. 2021). These results showed that hippocampal subfield asymmetry in the pediatric period continued similarly in adulthood.

We evaluated the hippocampal region as normal radiologically due to the 3.67% total hippocampal mean asymmetry rate (Wang et al. 2017). We also did not detect significant differences in hippocampal subfield lateralization across developmental periods. Steve et al. reported that different volumetric changes in different subfields of the hippocampal atrophy caused asymmetry in hippocampal sclerosis (HS) (Steve et al. 2014). Additionally, Moghaddam et al. reported that although they found atrophy in all areas of the ipsilateral hippocampus in left and right medial temporal lobe epilepsy (MTLE) patients compared to controls, the subiculum remained unchanged (Moghaddam et al. 2021). Therefore, we think that the hippocampal subfield asymmetry data we presented in this study can be used for comparison purposes in studies conducted in developmental periods.

### What are the possible factors that could give rise to nonlinear models of hippocampal subfields?

The nonlinear volumetric models observed in the development of hippocampal subfields are the macroscopic reflection of a complex, dynamic, and overlapping series of microscopic events occurring at the cellular level. The sigmoid, quadratic, and cubic models identified in our study can be linked to the sequential and overlapping dominance of processes like synaptogenesis, dendritic branching, glial cell proliferation, myelination, and synaptic pruning.

The initial, rapid volumetric expansion observed across all subfields in both sexes during early childhood (the ascending phase of the curves in Fig. 3) is a direct reflection of fundamental neuronal growth processes. This period is dominated by an exuberant wave of synaptogenesis and dendritic arborization, which dramatically increases the volume of the neuropil as neurons establish an overabundance of connections (Petanjek et al. 2011). This process of creating complex new circuitry is metabolically and structurally supported by the concurrent proliferation of glial cells, particularly astrocytes, which are integral to synapse formation and function (Allen and Eroglu 2017). In the CA4-dentate gyrus (DG) subfield specifically, this growth is uniquely augmented by sustained postnatal neurogenesis, a process now confirmed to persist in the human hippocampus throughout life, albeit at a declining rate with age (Boldrini et al. 2018). The quadratic model observed for the CA4-DG is likely the composite result of this cell addition combined with the maturation of existing cells.

The divergence from a common growth model into distinct, sex-specific trajectories during late childhood and adolescence strongly implicates the activational effects of gonadal hormones during puberty (Sisk and Foster 2004). The hippocampus is densely populated with receptors for androgens and estrogens, rendering it highly sensitive to the pubertal hormonal surge (McEwen 2001). This hormonal signaling acts upon the foundational circuits established in childhood, initiating a profound period of sex-specific reorganization.

In males, the consistent sigmoidal model observed for the total hippocampus and CA1-3 subfields (Fig. 3a and d) maps precisely onto the timeline of the pubertal testosterone surge (Wierenga et al. 2018). This powerful, time-limited hormonal event drives a distinct growth spurt that subsequently plateaus as adult hormonal levels are achieved, perfectly mirroring the sigmoidal model.

Conversely, the more complex quadratic and cubic models observed in females reflect a fundamentally different hormonal milieu. Female puberty is characterized not by a single surge, but by the onset of cyclical estrogen fluctuations (Grumbach 2002). Estradiol is a potent modulator of hippocampal plasticity, dynamically increasing dendritic spine density in regions like CA1 (Woolley and McEwen 1992). This continuous, cyclical remodeling is so pronounced that hippocampal volume has been shown to fluctuate across the human menstrual cycle (Protopopescu et al. 2008). This ongoing, dynamic influence, rather than a single pubertal event, provides a strong biological rationale for a more protracted and multi-phasic maturational curve, as captured by our quadratic and cubic models (Herting et al. 2012).

The plateauing of the curves in late adolescence for both sexes represents a final phase of maturation dominated by circuit refinement. This stabilization of volume is driven by large-scale synaptic pruning, an experience-dependent process where unnecessary or inefficient synapses are eliminated to optimize neural processing (Paus et al. 2008). This reduction in synaptic density counterbalances any minor volumetric increases from ongoing microstructural changes. Concurrently, the protracted process of myelination continues within hippocampal pathways, increasing the speed and efficiency of information transfer (Lebel and Deoni 2018).

Finally, the distinction between absolute and relative volume models (Fig. 4) is critical; while absolute volume reflects the intrinsic development of the hippocampus, the often flatter or even declining relative volume curves highlight its maturation in the context of the entire brain, where the rapid myelination of white matter tracts significantly increases the total intracranial volume throughout adolescence (Lebel and Deoni 2018).

Clinical importance of immature hippocampal subfields.

Various studies have reported that the plasticity of the hippocampus is associated with different effects (Erickson et al. 2011; Woollett and Maguire 2011; Lui et al. 2013; Yu et al. 2018). Various studies investigating childhood neuropsychiatric diseases (posttraumatic stress disorder, autism, schizophrenia, major depressive disorder, and Attention-deficit/hyperactivity disorder) reported that these diseases cause changes in the total volume and subfields of the hippocampus (Schumann et al. 2004; Felmingham et al. 2009; Van Erp et al. 2015; Schmaal et al. 2015; Wang et al. 2018; Li et al. 2023). Teicher et al. found that children exposed to childhood maltreatment had an average 6% volume reduction in CA2/CA3 and CA4/DG compared to those who did not experience childhood trauma (Teicher et al. 2012). MTLE is less common in children than adults (Cersósimo et al. 2011). Correctly describing changes in hippocampal subfields is also important in identifying MTLE in young children. Changes in histological subfields of the hippocampus have been reported when defining MTLE subtypes with HS (Blümcke et al. 2012). It has been reported that neuronal loss in HS is highest in CA1, moderate in CA3 and CA4, and least in CA2 (Steve et al. 2014). This neuronal loss may lead to hippocampal atrophy and thus volumetric changes in hippocampal subfields (Steve et al. 2014; Moghaddam et al. 2021). For these reasons, normal volumetric data from hippocampal subfields may help determine the localization of the seizure onset zone (abnormal lateralization) and evaluate hippocampal changes in neuropsychiatric diseases.

### Limitations

An important limitation of the present study is its cross-sectional design. Although cross-sectional analyses can detect age-related differences between individuals, they cannot capture within-subject developmental changes. As emphasized by Keresztes et al., cross-sectional associations in hippocampal subfields may differ from longitudinal trajectories; our results can be interpreted as reflecting between-subject differences rather than true developmental change (Keresztes et al. 2022). However, MRI acquisition in childhood is a challenging experience for both the child and the parents. Additionally, the imaging costs of large pediatric cohorts are very burdensome. Retrospective cross-sectional studies rule out these negative aspects. Additionally, the fact that the atlas in the volBrain HIPS segmentation protocol used in this study differed from that in other studies may have affected the results. Participants in this study consisted of a cohort of patients who underwent a control MRI to exclude headache and epileptogenic pathology. However, this group of patients represents the patient population most likely to undergo an MRI scan. Pediatric participants did not have headache diagnoses known to cause changes (gray matter increase) in the hippocampal region, such as migraine and cluster headache (Yang et al. 2018; Zhang et al. 2022). Additionally, none of the patients in this study who underwent MRI to exclude epileptogenic pathology had a diagnosis of temporal lobe epilepsy or hippocampal sclerosis, which causes changes (gray matter decrease) in the hippocampal region (Steve et al. 2014; Zheng et al. 2018). Although the participants in this study had normal hippocampal radiological anatomy, this should be considered when interpreting the results because the hippocampal region is more sensitive than other brain regions. Another important limitation of the present study concerns the spatial resolution of the MRI data. The volumetric analyses were performed on structural T1-weighted images with an isotropic voxel size of 1 mm³. Although this resolution is commonly used in clinical and research settings, several studies have emphasized that it may not provide sufficient detail to accurately delineate small hippocampal subfields. In particular, Wisse et al. highlighted that 1 mm isotropic scans can lead to partial volume effects and boundary ambiguities between adjacent subfields, potentially reducing the reliability and anatomical precision of hippocampal subfield volumetry (Wisse et al. 2021). The Kulaga-Yoskovitz atlas we used in this study did not directly segment the hippocampal internal structures (e.g., stratum radiatum lacunosum moleculare) below 1 mm. However, partial volume effects can be observed in voxels within the submillimetric hippocampal border regions at this resolution. Data for this study include only the Turkish pediatric population. Studies with various samples must determine whether these findings can be generalized to other child populations.

To overcome current limitations, future studies should use longitudinal designs to capture individual hippocampal development better and include broader, non-clinical pediatric samples to enhance generalizability. Employing higher-resolution and multimodal MRI protocols, such as submillimetric T2-weighted imaging, will allow for more anatomically precise segmentation of hippocampal subfields and reduce partial volume effects. Lastly, research involving ethnically and culturally diverse populations is needed to assess the broader applicability of findings.

## Conclusions

This study examined theage-related differences, sexual dimorphism, and asymmetry of the subfields of the hippocampus in the 2–18 age group in 4 different developmental periods and presented it with a different method than other studies. Unlike other studies, we contributed to this gap in the literature by characterizing the age-related volumetric differences of hippocampal subfields between the ages of 2 and 4. Overall, these findings revealed that the age-related differences of hippocampal structures showed heterogeneity based on sex, age, side, and subfield, with some subfields (CA4-DG) being less affected by age. Future research on hippocampal subfields in childhood and adolescence should consider the factors contributing to heterogeneity among subfields. Furthermore, how these factors relate to cognitive and neuropsychological outcomes should be examined.

**Table 1:** Descriptive statistics of age and total intracranial volume of 443 individuals aged 2–18 years according to developmental periods

Variable	Developmental period	Period comparisons			Sex comparisons				p
		Female + male		Tukey HSD (*p* < 0.0083)	Female		Male		
		Mean	SD (±)		Mean	SD (±)	Mean	SD (±)	
Age (year)	Toddler (T)	2.00	0.00	P, S, A	2.00	0.00	2.00	0.00	1.00
	Preschool (P)	3.83	0.82	T, S, A	3.85	0.77	3.81	0.85	0.796
	School age (S)	8.40	1.92	T, P, A	8.48	1.93	8.34	1.92	0.671
	Adolescence (A)	15.46	1.68	T, P, S	15.34	1.60	15.59	1.78	0.427
	2–18 age group	8.21	5.17	–	8.58	5.34	7.90	5.01	0.172
Age (month)	Toddler (T)	29.98	3.55	P, S, A	29.89	3.89	30.06	3.25	0.856
	Preschool (P)	51.98	10.33	T, S, A	52.02	9.34	51.95	11.04	0.967
	School age (S)	107.16	23.28	T, P, A	108.04	23.61	106.59	23.18	0.719
	Adolescence (A)	193.13	20.66	T, P, S	191.39	19.30	195.13	22.13	0.325
	2–18 age group	105.05	62.75	–	109.32	64.76	101.53	60.95	0.194
Total intracranial volume (cm^3^)	Toddler (T)	1183.20	115.18	P, S, A	1128.52	94.05	1235.93	110.19	**0.000**
	Preschool (P)	1268.58	125.32	T, S, A	1216.35	117.19	1305.98	118.01	**0.000**
	School age (S)	1368.11	142.14	T, P	1289.48	109.12	1419.91	137.98	**0.000**
	Adolescence (A)	1380.06	135.62	T, P	1317.79	111.68	1451.22	125.85	**0.000**
	2–18 age group	1319.85	149.18	–	1257.43	127.08	1371.23	146.62	**0.000**

Descriptive statistics were given as mean and standard deviation (SD). We compared variables belonging to developmental periods using the Post Hoc Tukey HSD test. According to the results of this test, we denoted the developmental period compared to the other periods that showed significant differences with their initials. The statistical significance level was determined as *p* < 0.0083 (Bonferroni-corrected). Variables that reached statistical significance are indicated with a bold character in the table

**Table 2:** Descriptive statistics of total hippocampal and hippocampal subfields across age ranges 2–18 years and developmental periods, independent of sex

Variable	Developmental Period	Absolute volume (cm^3^)		Multiple comparisons (Tukey HSD) *p* < 0.0083	Relative volume (%)		Multiple comparisons (Tukey HSD) *p* < 0.0083	Asymmetry (%)		Multiple comparisons (Tukey HSD) *p* < 0.0083
		Mean	SD (±)		Mean	SD (±)		Mean	SD (±)	
Total hippocampal volume	Toddler (T)	5.503	0.549	P, S, A	0.466	0.034	A	2.739	4.089	n.s.
	Preschool (P)	6.129	0.726	T, S, A	0.484	0.042	n.s.	3.825	4.551	n.s.
	School age (S)	6.614	0.785	T, P	0.484	0.040	n.s.	3.553	3.943	n.s.
	Adolescence (A)	6.849	0.759	T, P	0.498	0.045	T	4.073	4.347	n.s.
	2–18 age group	6.401	0.853	–	0.486	0.042	–	3.671	4.257	–
Total CA1-3 volume	Toddler (T)	3.340	0.384	P, S, A	0.283	0.024	P, S, A	6.742	7.615	n.s.
	Preschool (P)	3.807	0.528	T, S, A	0.300	0.032	T	7.240	7.673	n.s.
	School age (S)	4.127	0.537	T, P	0.302	0.028	T	6.403	6.876	n.s.
	Adolescence (A)	4.243	0.547	T, P	0.308	0.033	T	7.682	7.003	n.s.
	2–18 age group	3.969	0.595	–	0.301	0.031	–	7.032	7.233	–
Total CA4-DG volume	Toddler (T)	0.600	0.097	S, A	0.051	0.008	n.s.	0.626	11.993	n.s.
	Preschool (P)	0.647	0.105	A	0.051	0.008	n.s.	0.106	11.693	n.s.
	School age (S)	0.677	0.122	T	0.050	0.009	n.s.	1.410	11.575	n.s.
	Adolescence (A)	0.703	0.119	T, P	0.051	0.009	n.s.	0.366	11.715	n.s.
	2–18 age group	0.666	0.118	–	0.051	0.009	–	0.656	11.672	–
Total subiculum volume	Toddler (T)	1.563	0.207	S, A	0.132	0.015	n.s.	– 4.848	9.099	n.s.
	Preschool (P)	1.676	0.220	S, A	0.132	0.014	n.s.	– 2.523	8.741	n.s.
	School age (S)	1.810	0.265	T, P	0.132	0.015	A	– 2.569	9.399	n.s.
	Adolescence (A)	1.902	0.222	T, P	0.138	0.014	P, S	– 2.642	7.401	n.s.
	2–18 age group	1.766	0.260	–	0.134	0.015	–	– 2.859	8.671	–

Descriptive statistics were given as mean and standard deviation (SD). Hippocampal relative volume values ​​are the ratio of the relevant hippocampal subfield to the total intracranial volume. We compared variables belonging to developmental periods using the Post Hoc Tukey HSD test. According to the results of this test, we denoted the developmental period compared to the other periods that showed significant differences with their initials. We used the Bonferroni-corrected significance value as 0.0083. *n.s.* non-significant (*p* > 0.0083)

**Table 3:** Comparison of absolute and relative age-related differences in the total hippocampus and hippocampal subfields between females and males in the pediatric period, and the variables related to these models

Volume measurements		Model summary							
		Female (n:200)				Male (n:243)			
		Age-related model	R^2^	F	p	Age-related model	R^2^	F	p
Total (bilateral) hippocampus	Absolute (cm^3^)	Quadratic	0,273	36,943	**< 0.001**	Sigmoid	0,327	116,983	**< 0.001**
	Relative (%)	Quadratic	0,030	3,044	**0.049**	Inverse	0,070	18,056	**< 0.001**
Right hippocampus	Absolute (cm^3^)	Quadratic	0,272	36,800	**< 0.001**	Sigmoid	0,333	120,545	**< 0.001**
	Relative (%)	Quadratic	0,035	3,621	**0.029**	Inverse	0,076	19,942	**< 0.001**
Left hippocampus	Absolute (cm^3^)	Quadratic	0,258	34,300	**< 0.001**	Sigmoid	0,302	104,413	**< 0.001**
	Relative (%)	**–**	**–**	**–**	n.s.	Inverse	0,056	14,247	**< 0.001**
Total (bilateral) CA1-3	Absolute (cm^3^)	Sigmoid	0,248	65,329	**< 0.001**	Sigmoid	0,302	104,379	**< 0.001**
	Relative (%)	Logarithmic	0,028	5,679	**0.018**	Sigmoid	0,069	17,984	**< 0.001**
Right CA1-3	Absolute (cm^3^)	Sigmoid	0,238	61,719	**< 0.001**	Sigmoid	0,308	107,477	**< 0.001**
	Relative (%)	Linear	0,027	5,460	**0.020**	Sigmoid	0,072	18,645	**< 0.001**
Left CA1-3	Absolute (cm^3^)	Sigmoid	0,234	60,525	**< 0.001**	Sigmoid	0,259	84,153	**< 0.001**
	Relative (%)	Inverse	0,025	5,176	**0.024**	Sigmoid	0,052	13,220	**< 0.001**
Total (bilateral) CA4-DG	Absolute (cm^3^)	Quadratic	0,073	7,774	**0.001**	Cubic	0,101	8,923	**< 0.001**
	Relative (%)	**–**	**–**	**–**	n.s.	**–**	**–**	**–**	n.s.
Right CA4-DG	Absolute (cm^3^)	Cubic	0,071	4,975	**0.002**	Cubic	0,099	8,769	**< 0.001**
	Relative (%)	**–**	**–**	**–**	n.s.	**–**	**–**	**–**	n.s.
Left CA4-DG	Absolute (cm^3^)	Quadratic	0,063	6,628	**0.002**	Cubic	0,088	7,651	**< 0.001**
	Relative (%)	**–**	**–**	**–**	n.s.	**–**	**–**	**–**	n.s.
Total (bilateral) subiculum	Absolute (cm^3^)	Cubic	0,231	19,640	**< 0.001**	Cubic	0,239	24,989	**< 0.001**
	Relative (%)	Cubic	0,081	5,734	**0.001**	Power	0,041	10,315	**0.001**
Right subiculum	Absolute (cm^3^)	Cubic	0,229	19,459	**< 0.001**	Sigmoid	0,207	62,903	**< 0.001**
	Relative (%)	Cubic	0,073	5,162	**0.002**	Power	0,032	8,014	**0.005**
Left subiculum	Absolute (cm^3^)	Cubic	0,197	16,030	**< 0.001**	Power	0,236	74,442	**< 0.001**
	Relative (%)	Cubic	0,079	5,614	**0.001**	Power	0,039	9,856	**0.002**

Hippocampal relative volume values are the ratio of the relevant hippocampal region to the total intracranial volume. The statistical significance level was determined as *p* < 0.05. Variables that reached statistical significance in total hippocampal and hippocampal subfield volumes are shown in bold in the table. Graphs of the curves (age-related model) that best illustrate age-related relationships in scatter plots created in SPSS for all hippocampal subfields are presented in Figs. 3 and 4. *CA* cornu ammonis, *DG* dentate gyrus, *n.s.* non-significant (*p* > 0.05)

## Data Availability

No datasets were generated or analysed during the current study.
